# Deuterium Adsorption on Free-Standing Graphene

**DOI:** 10.3390/nano11010130

**Published:** 2021-01-08

**Authors:** Mahmoud Mohamed Saad Abdelnabi, Chiara Izzo, Elena Blundo, Maria Grazia Betti, Marco Sbroscia, Giulia Di Bella, Gianluca Cavoto, Antonio Polimeni, Isabel García-Cortés, Isabel Rucandio, Alejandro Moroño, Kailong Hu, Yoshikazu Ito, Carlo Mariani

**Affiliations:** 1 Dipartimento di Fisica, Sapienza Università di Roma, P.le Aldo Moro 2, 00185 Rome, Italy; mahmoud.abdelnabi@uniroma1.it (M.M.S.A.); izzo.1704441@studenti.uniroma1.it (C.I.); marco.sbroscia@uniroma1.it (M.S.); giuliadibella05@gmail.com (G.D.B.); antonio.polimeni@roma1.infn.it (A.P.); 2 Dipartimento di Fisica and INFN Sezione di Roma 1, Sapienza Università di Roma, P.le Aldo Moro 2, 00185 Rome, Italy; maria.grazia.betti@roma1.infn.it (M.G.B.); gianluca.cavoto@roma1.infn.it (G.C.); 3 CIEMAT, Avenida Complutense 40, 28040 Madrid, Spain; isabel.garciacortes@ciemat.es (I.G.-C.); isabel.rucandio@ciemat.es (I.R.); alejandro.morono@ciemat.es (A.M.); 4 Institute of Applied Physics, Graduate School of Pure and Applied Sciences, University of Tsukuba, Tsukuba 305-8573, Japan; kailong_hu@hotmail.com (K.H.); ito.yoshikazu.ga@u.tsukuba.ac.jp (Y.I.)

**Keywords:** nano porous graphene, deuterium, graphane, XPS, UPS, Raman

## Abstract

A suitable way to modify the electronic properties of graphene—while maintaining the exceptional properties associated with its two-dimensional (2D) nature—is its functionalisation. In particular, the incorporation of hydrogen isotopes in graphene is expected to modify its electronic properties leading to an energy gap opening, thereby rendering graphene promising for a widespread of applications. Hence, deuterium (D) adsorption on free-standing graphene was obtained by high-energy electron ionisation of D${}_{2}$ and ion irradiation of a nanoporous graphene (NPG) sample. This method allows one to reach nearly 50 at.% D upload in graphene, higher than that obtained by other deposition methods so far, towards low-defect and free-standing D-graphane. That evidence was deduced by X-ray photoelectron spectroscopy of the C 1s core level, showing clear evidence of the D-C sp${}^{3}$ bond, and Raman spectroscopy, pointing to remarkably clean and low-defect production of graphane. Moreover, ultraviolet photoelectron spectroscopy showed the opening of an energy gap in the valence band. Therefore, high-energy electron ionisation and ion irradiation is an outstanding method for obtaining low defect D-NPG with a high D upload, which is very promising for the fabrication of semiconducting graphane on large scale.

## 1. Introduction

One of the most pursued key goals in graphene functionalisation is the achievement of a two-dimensional (2D) material with unvaried mechanical strength and stiffness but with different and tailored electronic and transport properties. In fact, while the extremely high mobility of graphene renders it appealing for applications, its semi-metallic nature represents a hindrance. Graphane is the forecasted semiconducting [1,2,3] form of graphene, where the carbon (C) atoms in the honeycomb lattice are bonded to H atoms. Hence, the C–C sp${}^{2}$ bonds of graphene are modified towards sp${}^{3}$ H–C bonds in graphane [1]. Graphane and nano-structured/functionalised graphene may constitute innovative systems for designing highly efficient electronic devices, thanks to the remarkable mechanical robustness and flexibility typical of 2D materials [4,5,6], coupled with a semiconducting character [7,8,9,10], unlike graphene. Recently, graphane as constituted by tritium (T)—the β-unstable isotope of hydrogen—covalently bonded to graphene, has been also suggested to represent an ideal candidate for the realisation of a new detector able to measure the endpoint of the tritium β-electron spectrum. This prototypical T-graphene-based detector (the Ptolemy project [11]) has been designed to improve the electron neutrino mass measurement, thanks to its very high sensitivity. A scaled version of such a T-graphene-based detector would in principle be able to measure the cosmological neutrino background generated a few seconds after the big bang [12]. The stabilisation of T is a crucial condition for the detector, which could be achieved by T chemisorption on graphene [11]. Since T presents severe safety issues, fundamental tests on the adsorption can be carried out with lighter tritium isotopes—deuterium and hydrogen. In fact, H and D are expected to have very similar chemical reactivity to tritium with the C atoms in the graphene mesh, without presenting any radio-protection limitations.

All these potential outcomes of graphane share the need to incorporate significant hydrogen (or its isotopes) quantities in the carbon mesh. Several experiments have been carried out till now, mainly using hydrogen as the element to functionalise graphene, which also presents very similar chemical reactivity to that of deuterium. Graphene hydrogenation can be achieved by different methods, ranging from hot [13,14,15] and cold plasma [16] deposition, to molecular H${}_{2}$ high-temperature cracking [17,18]; the adsorption was done on a variety of graphene samples, from exfoliated layers [15,19], to chemical-vapour-deposition (CVD) grown flakes [13,20,21], to metal-supported graphene [17,18,22,23,24]. The H uptake by graphene in these works presents different saturation values, with an upper limit of 25% [18], probably depending on the different graphene morphology (substrate-supported, transferred flakes, etc.) and hydrogenation method. The uptake has been most often evaluated by the modified intensity of the Raman features with respect to pristine graphene, while the presence of the sp${}^{3}$ bond was only seldom actually verified through direct measurement of the C 1s lineshape. Although Raman measurements can represent a powerful technique in characterising graphene, it is not generally possible to distinguish the effect of hydrogen bonded to graphene from the presence of defects induced in the lattice during the hydrogen incorporation process.

The upper limit for hydrogen (deuterium) uploading can be due both to the presence of defects/edges (in 2D graphene flakes) or to the influence of the substrate (in metal-supported graphene). To overcome these drawbacks we propose to expose to deuterium high-quality, three-dimensional (3D), warped, free-standing graphene. Here, we study the effect of deuterium incorporation in nanoporous graphene samples: NPG is constituted by a compact, bi-continuous interconnected 3D arrangement of high-quality graphene veils, with 1000 m${}^{2}$/g specific surface area, high transport properties and Dirac fermion [25,26], composed by one to a few layers, weakly interacting among each other [27,28]. The veils present a curved structure at the sub-m scale, with intrinsic local scale curvatures and rippling that favour H and D adsorption. In fact, while H and D approach the C mesh, chemisorption is favoured by an energy barrier decrease [29] associated with the pull out of the C atom towards the proton or deuteron to form the sp${}^{3}$ bond [30]. Thus, in comparison to conventional 2D graphene, the use of NPG to adsorb H and D results is of great advantage to obtain high-quality and free-standing graphane. We recently established the uptake of hydrogen and deuterium as adsorbed on NPG by low-energy ion deposition [31], by measuring the clear presence of the sp${}^{3}$ bonds by X-ray photoelectron spectroscopy (XPS) in the chemically shifted component of the C 1s core level. The lattice quality was determined by the relatively low defect band in the Raman spectra, showing achievement of high-quality free-standing graphane with a maximum uptake of 25 at.% (H-NPG) and 36 at.% (D-NPG), and experimentally demonstrating the analogous chemical bond with the C atoms. In view of the importance of carefully characterising the heavier isotopes, towards eventually use T, we focus here on a different deposition method using medium-energy deuteron ions excited by high-energy electrons.

In this paper, after characterising and describing how to prepare a properly clean NPG sample, we describe its direct exposure to a deuterium ion beam into a deuterium atmosphere, with the ions generated by the irradiation of the gas with 1.8 MeV electrons; the D${}^{+}$ ions are then drifted with an applied electric field towards the graphene sample, to be deuterium loaded. With Raman spectroscopy, we demonstrate the very high quality of the obtained D-graphane. We directly measured the sp${}^{3}$ component in the C 1s core level by XPS. We show the fingerprint of achieved D–C bond formation and show that we can reach about 50 at.% deuterium upload adsorbed by NPG, an amount even higher than that previously obtained by using low-energy ions [31]. Furthermore, we show that deuterium causes quenching of the electronic spectral density below the Fermi level, confirming the semiconducting nature of graphane. The present results on D-NPG constitute a further step towards the elaboration of a strategy for perspective atomic T-graphane growth for new prototype detectors [11].

## 2. Materials and Methods

Nanoporous graphene was synthesised by using a nano-porous Ni-based chemical vapour deposition (CVD) method [25,32,33,34,35]. First, we prepared Ni${}_{30}$Mn${}_{70}$ ingots by melting both pure metals (purity > 99.9 at.%) in an Ar-protected arc melting furnace. After annealing as-prepared Ni${}_{30}$Mn${}_{70}$ ingots at 900 ${}^{\circ }$C for 24 h for achieving the microstructure and composition homogeneity, the ingots were cold-rolled to 50 m-thin sheets at room temperature (RT, 25 ± 2 ${}^{\circ }$C). In a second step, nanoporous Ni was obtained from the Ni${}_{30}$Mn${}_{70}$ sheet by using the chemical dealloying in a 1.0 M (NH${}_{4}$)${}_{2}$SO${}_{4}$ aqueous solution for 12 h at 50 ${}^{\circ }$C. Having obtained nano-porous Ni, the samples were washed out with distilled water, and then with ethanol; after, they were dried in vacuo. The nanoporous Ni substrates were washed, loaded on a corundum plate, put into the centre of a quartz tube (ϕ30×ϕ30 × 1000 mm) furnace and annealed at 900 ${}^{\circ }$C under flowing gas mixed by 200 sccm Ar (purity 99.999%) and 100 sccm H${}_{2}$ (purity 99.999%) for 3 min as reduction pre-treatments. After that, benzene (0.5 mbar, 99.8%, anhydrous, Sigma Aldrich) as a source of C atoms for the CVD process was introduced, together with gas flow of Ar (200 sccm) and H${}_{2}$ (100 sccm), for graphene growth at 800 ${}^{\circ }$C for 120 s. The furnace was then immediately opened and the the quartz tube was quickly cooled with a fan to RT. The so-obtained nanoporous Ni substrates were dissolved by using a solution (1.0 M) of HCl at 25 ${}^{\circ }$C for 12 h; then they were transferred into another HCl solution (2.0 M) to fully remove residual Ni and Mn as much as possible at 60 ${}^{\circ }$C. The NPG samples were repeatedly washed by distilled water and kept in water for one day, then transferred into isopropanol (99.7%, Kanto Chemical Co. Inc., Tokyo, Japan) and kept for one week. Finally, the 3D porous graphene sheets were dried with a standard supercritical drying method using CO${}_{2}$ gas (purity: 98%).

**Figure 1:**
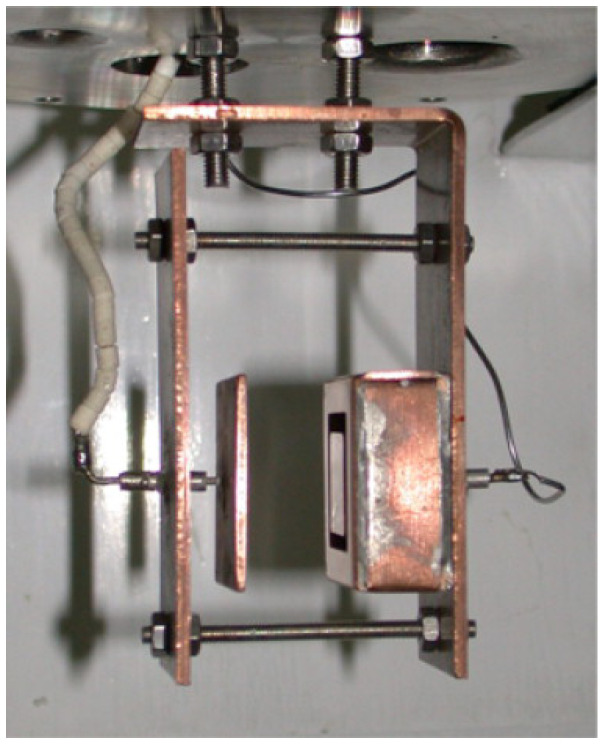
Picture of the sample holder provided with copper electrodes in order to induce deuterium ion loading by an applied electric field.

Deuteration of NPG was achieved at the CIEMAT laboratory in Madrid [36]. This installation is mainly devoted to experiments of radiation effects in deuterium absorption, desorption and permeation in fusion materials [37,38,39]. Electric field-induced deuterium loading of graphene was carried out while making use of a Van de Graaff electron accelerator to ionise the deuterium gas, with the NPG sample mounted into a cell (shown in Figure 1) provided with two copper electrodes: one was grounded and the other one polarised with −500 volts during the experiment; the NPG sample was mounted onto the negative electrode. The cell was introduced into a sealed chamber filled with deuterium gas at 1 bar.

This chamber was mounted in the beam line of a Van de Graaff electron accelerator. A 0.05 mm aluminium foil separated the gas chamber and the accelerator beam line in vacuum. In this way, 1.8 MeV electrons passed through the aluminium foil into the gas chamber. Once in the gas chamber, the 1.8 MeV electron beam was collimated in such a way that it passed between the two copper electrodes without touching the electrodes or the sample, just ionising the deuterium gas between the two electrodes without irradiating the sample. Once the deuterium gas was ionised by the 1.8 MeV electron beam, due to the action of an electric field perpendicular to the beam, free electrons generated by the ionising beam drifted to the grounded electrode and deuterium positive ions were directed to the negative (−500 V) electrode, where the NPG sample was placed. The ion density current reaching the graphene sample was measured during the experiment, being 1.4 mA/cm${}^{2}$, and the sample was exposed to this ion current for 4 h. We estimated a final exposure of ≤1.3 × 10${}^{20}$ D${}^{+}$ ions loaded in the actual NPG sample. In this experiment the role of the 1.8 MeV electron beam was just to ionise the deuterium gas without irradiating the sample. Due to the pressure of the deuterium gas, the ions drifted to the graphene could not reach enough energy to produce any damage to the graphene sample.

After deuteration, the D-NPG sample was eventually transferred in dry ambient conditions to the XPS chamber.

The XPS and ultraviolet photoelectron spectroscopy (UPS) experiments were performed at Sapienza University in Rome, in the Nanostructures at Surfaces laboratory. The UHV chambers had a base pressure in the high 10${}^{-11}$ mbar range. Mg K${}_{\alpha }$ radiation (hν = 1253.6 eV) was used as the X-ray source (PSP TA10); emitted photoelectrons were analysed with a hemispherical VG Microtech Clam-2 electron electrostatic analyser in constant pass energy (PE) mode set at 50 eV. The overall energy resolution was ≤1 eV—further details in [40,41,42,43]. Clean gold foil in electrical contact with the sample was used for calibrating the Fermi level (Au 4f${}_{7/2}$ core-level at 84.0 eV BE). The D-NPG sample was measured firstly as-irradiated, then again after a sequence of annealing steps, as discussed later. The UPS data have been taken with a Gammadata VUV 5000 microwave excited monochromatised He source, with He${}_{I\alpha }$ radiation (21.218 eV). Photoelectrons were analysed with an electrostatic hemisferical Scienta SES 200 analyser equipped with a multi-channel-plate detector, operated with an overall 20 meV energy resolution.

The Raman experiments were carried out at the Optical Spectroscopy of Nanostructured Materials laboratory, at Sapienza University of Rome. As the excitation source, a Nd:YVO4 laser at 532 nm (DPSS series by Lasos) was employed. The measurements were acquired in a backscattering configuration, by focusing the laser and collecting the signal with an Olympus 50× objective (NA = 0.50). The laser power was kept below 100 W measured at the entrance of the objective to avoid sample damage, having checked that no changes were induced in the spectra with such a power, and that powers higher by about one order of magnitude were needed to induce possible damages. A 200-mm focal length monochromator (Isoplane 160) equipped with a 150 or 300 grooves/mm grating was employed to spectrally analyse the Raman signal, which was detected by a back-illuminated Si CCD Camera (model 100BRX by Princeton Instruments). Rejection for elastically scattered laser light has been achieved by a sharp high-pass Razor edge filter at 535 nm (Semrock).

Scanning electron microscopy (SEM) images of NPG at the μm spatial scale were obtained at the CNIS laboratory of Sapienza University, using a field-emission Zeiss Auriga 405 instrument with a nominal resolution of 1 nm at maximum magnification and a beam energy of 9.5 keV, at a working distance of ∼3.5 mm.

## 3. Results and Discussion

In order to overcome the limitations in hydrogen/deuterium incorporation, a cleaning protocol should be adopted, so to minimise defects and contamination of NPG samples. In fact, both the synthesis and the deuterium upload procedures, despite being carried out in controlled and the cleanest possible conditions, led to slight yet unavoidable oxygen contamination. A proper preparation procedure of the pristine NPG sample before exposure to deuterium can be achieved by annealing at 600 ${}^{\circ }$C in an ultra-high vacuum for several hours; that guarantees clean, high-quality free-standing graphene, apt to be functionalised. In the following, we first describe the NPG preparation and cleaning procedures; then we study by Raman and photoemission spectroscopy the D-adsorbed NPG achieved by bombardment with medium-energy ions.

### 3.1. NPG Sample Preparation Prior to D-Exposure

**Figure 2:**
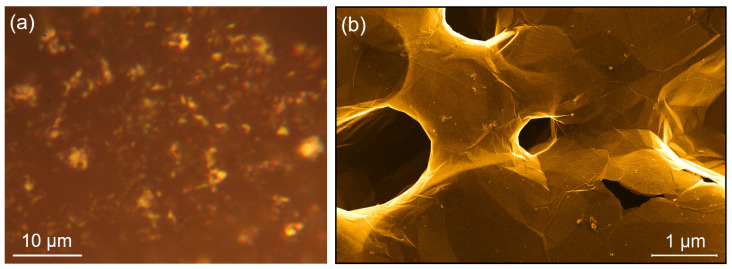
(**a**) Optical image of the NPG sample acquired with a 100× magnitude objective. (**b**) Scanning electron microscopy (SEM) image of the NPG sample, showing the presence of pores several hundreds of nanometres in size and the continuous structure of the graphene veil.

The NPG sample microscopically appears with an apparent non-homogeneous texture by optical microscopy (Figure 2a), while the SEM image reveals a three-dimensional compact graphene sample formed by bi-continuous veils without frayed edges, with a porous structure and 300–900 nm diameter pore size [25], as shown in Figure 2b.

As we will discuss in the following, the Raman spectra acquired in our NPG samples show a very slight presence of defect/disorder related peaks, typical of a very high-quality and low-defect one-to-few layer continuous graphene sheet with a turbostratic stacking, thereby having only weak bonding between the sheets, as discussed in previous papers [27,28].

**Figure 3:**
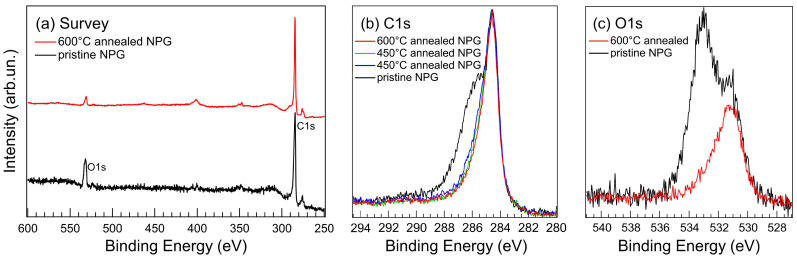
(**a**) XPS survey spectra of as-mounted pristine NPG and after annealing at 600 ${}^{\circ }$C temperature in UHV; (**b**) C 1s core level XPS spectra of NPG as pristine sample and as a function of annealing temperature and time, from the bottom 450 ${}^{\circ }$C for 5 h, 450 ${}^{\circ }$C for 8 h and 600 ${}^{\circ }$C for 30 min; (**c**) O 1s core level XPS spectra of NPG as pristine sample and after annealing at 600 ${}^{\circ }$C for 1 h.

NPG was measured by XPS on pristine samples and after an annealing procedure in UHV, aiming at reducing possible residual contamination. The C 1s and the O 1s core levels as functions of annealing temperature and time are displayed in Figure 3b,c. The pristine sample is characterised by the main C 1s peak at 284.6 eV characteristic of the covalent sp${}^{2}$ bond between adjacent C atoms in the graphene mesh, accompanied by a broad band roughly centred at ∼286 eV binding energy (BE) associated with multiple CO${}_{x}$ bonds [44,45,46], due to the residual contamination after sample synthesis.

Residual oxygen is the only intense contaminant present, with a concentration of ≥20 at.% estimated by taking into account the relative excitation cross sections of the O 1s and C 1s core levels [47], as shown in the survey spectrum in Figure 3a. The O 1s core level is composed by two main structures at 531.3 eV and 533.0 eV BE, characteristic of the C=O and C–O bonding, respectively [48,49]. Long annealing (several hours) at 450 ${}^{\circ }$C reduces the broad high-BE CO${}_{x}$ band in the C 1s structure, up to a final annealing at a higher temperature (600 ${}^{\circ }$C) that reduces it to <9 at.%. The corresponding O 1s residual contamination is overall reduced in concentration to a few percent and modified in composition, remaining dominant the C=O component. We remark that a higher annealing temperature, while it could possibly further reduce the C=O contamination, on the other hand would be detrimental because it would produce some graphene amorphisation [31]. This cleaning procedure in UHV is necessary to heavily reduce the contaminants and to eventually obtain the typical C 1s lineshape of clean graphene [31]. We remark that, once properly UHV-cleaned by annealing, the NPG C 1s core level does not regain the same intense CO${}_{x}$ band even after exposure to ambient pressure.

### 3.2. Raman Evidence of Low-Defected Graphane

Raman spectroscopy represents a non-invasive, all-optical technique particularly effective to probe the formation of new bonds with other atomic species, to achieve information on the number of layers, or to verify the presence of defects, doping, strain and lattice deformation in graphene [50,51,52] and other two-dimensional materials [53,54,55,56]. Previous Raman studies in transition-metal dichalcogenides revealed how energetic (few MeV) hydrogen-ion beams induce defects in the crystal matrix [57]; in contrast, low-energy beams (few eV) do not create defects, while the ions penetrate few layers, leading to the formation and accumulation of molecular hydrogen, and in turn to the creation of highly strained H${}_{2}$-filled domes [56,58,59]. In other layered materials, hydrogen can be incorporated in the crystal matrix with potential interest for hydrogen storage, and Raman measurements can be exploited to attest the hydrogen incorporation [54]. In the case of graphene, hydrogen ions can either bind to the carbon atoms or induce defects.

To have a comprehensive characterisation of the deuterated sample, Raman measurements were performed both before and after the D-exposure. Raman spectroscopy has been by far among the most effective and well used techniques for estimating graphene lattice quality [50]. The Raman spectrum of graphene is characterised by the G band—related to a first-order scattering process involving lattice modes with zero momentum (q=0) at the centre of the Brilllouin zone (Γ point)—and the 2D peak—related to a second-order process involving two phonons with opposite momentum (q,−q) near the K point [50]. Further modes related to second-order processes, namely, the D and D’ peaks, may originate in the presence of disorders, defects or following the formation of new bonds [50]. However, hydrogen bonding to graphene and the formation of defects have analogous effects on the Raman spectrum—both resulting in the appearance/increase of the D and D’ peak intensities—which makes it subtle to distinguish the latter from the former. In previous studies [13,14,16,60,61], remarkable increases in the D and D’ peaks were often directly interpreted as an evidence of the C–H bond formation, while our results show how this interpretation can be questionable.

**Figure 4:**
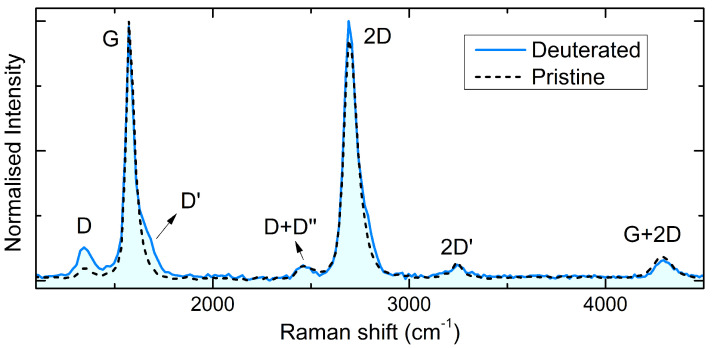
Raman spectrum of the deuterated NPG sample (blue line), acquired with a λ = 532.2 nm excitation laser. The Raman spectrum of the pristine sample (black dashed line) is shown for comparison, to emphasise the slight increases in the D and D’ peaks following the D-exposure.

In Figure 4, we compare the normalised Raman spectra of the deuterated sample (blue solid line) and of the pristine sample (black dashed line), to highlight the changes induced by the irradiation.

Both the G peak at 1578 cm${}^{-1}$ and the 2D peak at 2700 cm${}^{-1}$ are visible in the spectrum of our pristine sample and have comparable intensities, indicating a high quality of the sample. This is also confirmed by the low intensity of the D peak, at 1348 cm${}^{-1}$. Peaks related to higher order scattering process can also be seen (D+D”, 2D’, G+2D). After the deuteration, only minor changes can be seen in the spectrum, consisting of a very small intensity decrease of the 2D peak, and a modest increase of the D and D’ peak intensity. All the other higher order peaks remain nearly unchanged. Our Raman measurements clearly show that the high-quality of the graphene mesh is preserved after the high deuteration and the small increase of the defect-related peaks can be associated with the lattice distortion due to the deuterium incorporation. In the previous literature on H or D adsorption on graphene, the D/G Raman band intensity ratio is very much higher [13,14,16,60,61], suggesting how other hydrogenation/deuteration methods induce high density of other defects (vacancies, frayed borders, contaminants, etc.). Thus, one cannot interpret the only presence of defect-related peaks as a fingerprint of D incorporation, but rather a direct photoemission experiment can verify the chemical bonding and quantify the D upload.

### 3.3. Photoelectron Spectroscopy Fingerprints of D-NPG

**Figure 5:**
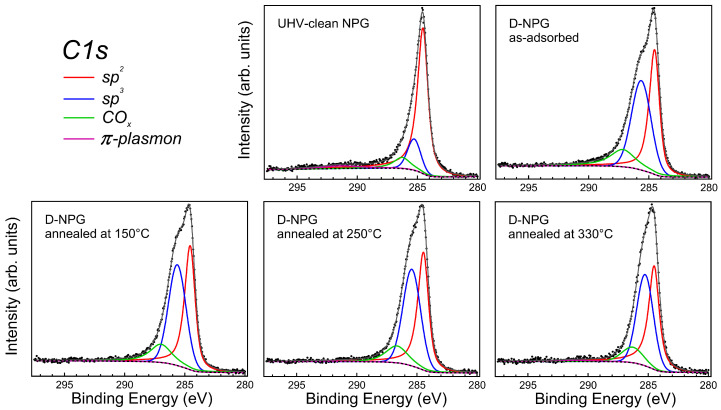
C 1s core level XPS spectra of UHV-clean NPG, as-adsorbed D-NPG and D-NPG after annealing at 150, 250 and 330 ${}^{\circ }$C: experimental data (black dots), sp${}^{2}$ fitting components (red lines), sp${}^{3}$ components (blue lines), CO${}_{x}$ components (green lines), π- plasmon excitation (pink lines), Shirley background (black dashed lines) and fitting sum curve (gray lines).

The C 1s core level spectra of the D-NPG sample as-adsorbed and after subsequent temperature annealing steps, along with the data of the UHV-clean one for comparison, are plotted in Figure 5.

The experimental data were fitted with components representing the different chemically shifted environments, after subtracting a Shirley background. In particular, we used the asymmetric Doniach–Sunjic (DS) lineshape for the main sp${}^{2}$ component (asymmetry parameter ≤ 0.1 [62,63,64]), where the asymmetry is due to the intrinsic semimetallicity of graphene; for all the other components, we used Gaussian–Lorentzian (Voigt lineshape) curves, taking into account both the intrinsic excitation linewidth (Lorentzian profile) and the overall experimental uncertainty (Gaussian profile). Fitting data are reported in Table 1.

The most prominent component of the C 1s spectra is associated with the sp${}^{2}$ bonding at 284.6 eV binding energy (BE). Aside from this main peak, the structure at about 285.3 eV BE in the clean NPG is associated with an sp${}^{3}$-like distorted bond. For the clean sample, this structure originates from the highly bent or wrinkled regions of the nano-pores, as recently discussed in micro-spectroscopy experiments [27,28]. There is another broad component characteristic of the π bonding in graphene at about 291.3 eV BE, thereby at 6.7 eV from the main peak, which is the plasmon associated with the π collective excitations [31,65,66,67]. Finally the component at about 286.3–287.0 eV is due to the residual C–O bond in the different possible CO${}_{x}$ configurations [44,45,46]. We underline that there is not any low-BE C 1s core-level component associated with unsaturated C dangling bonds at vacancy sites [10,23,68,69,70,71], not even in the deuterated sample, demonstrating the absolutely non-destructive deuteration of NPG.

Deuterium uptake induces the following changes in the C 1s lineshape: (i) increase of the sp${}^{3}$ component; (ii) quenching of the π plasmon excitation; (iii) increase of the CO${}_{x}$ component. The relative intensity of the sp${}^{3}$ component is a sign of bond deformation with respect to the pure π bonding, and thus of D–C chemical bonding when NPG is exposed to deuterium. The distortion of bonds introduced by the D loading is also reflected in the broadening of the sp${}^{3}$ component when compared to the clean NPG case (see Table 1). We can define Θ = I(sp${}^{3}$)/[I(sp${}^{2}$) + I(sp${}^{3}$)] as the relative ratio, and in the as-deposited D-NPG sample Θ reaches a value of 0.51 (±5%), corresponding to the fraction of D atoms bonded to C atoms in the graphene mesh. This value is higher than previously obtained in an experiment on NPG with free-standing graphene and low-energy ion deposition [31]. Another clear fingerprint of the bond distortion due to the D–C bonding is the quenching of π-plasmon, whose relative intensity reduces from ∼5.3% in the clean NPG to an almost negligible signal after D deposition. Since some residual oxygen contamination is present in the as-deposited D-NPG, we annealed the sample to increasingly higher temperatures up to 330 ${}^{\circ }$C, a safe value to avoid D desorption [31]. After this high-temperature annealing, the residual contamination is clearly reduced and the sp${}^{3}$-related peak at 285.3 eV BE sharpens; the reduction of the FWHM shows how spurious sp${}^{3}$ bonds introduced during the deuterium uploading are already amended at the moderate temperature of 150 ${}^{\circ }$C (see Table 1). Furthermore, the final Θ value remains stable at 0.49 (±5%), thereby corresponding to the unprecedented value of ∼50 at.% uptake of D bonded to the C atoms.

**Figure 6:**
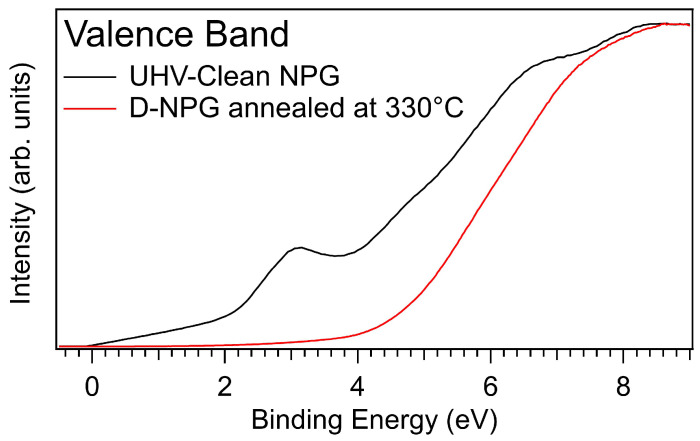
UPS valence band spectra (photon energy 21.218 eV) of UHV-cleaned NPG and of D-NPG annealed at 330 ${}^{\circ }$C.

The adsorption of H on graphene is known to turn graphene from a semimetal to a semiconductor [1,2,21,72]. Due to the very similar chemical bonding of D on the C atoms of graphene, the observation of the opening of an energy gap in the valence band of D-NPG would represent a further proof of the achievement of graphane. The valence band of D-NPG in comparison with that of clean NPG, excited with He${}_{I\alpha }$ radiation, is shown in Figure 6.

In the UHV-clean NPG we observe the typical electronic spectral density of graphene, characterised by (i) the linear behaviour towards the Fermi level (E${}_{F}$) reflecting the Dirac cone band dispersion, (ii) the peak at about 3 eV BE due to the π-state and (iii) the broad features between 6 and 8.5 eV BE associated with the σ−π band [27], evidencing the high quality of this free-standing graphene sample. The D-saturated NPG after annealing at 330 ${}^{\circ }$C presents a quenching of the π peak, a redistribution of the of σ−π states and the strong reduction of the spectral density of states below the Fermi level, with the top of the valence band at ∼3.8 eV from E${}_{F}$, in good agreement with what observed for H-decorated graphene [21].

The observed gap opening is further proof of the achievement of semiconducting graphane after chemisorption of D, in unprecedented, highly D-uploaded graphene with very high quality. We underline that the high percentage of sp${}^{3}$ bonds associated with D–C in semiconducting graphane is not reflected in a very high intensity of the Raman D band. This suggests how attention should be paid when interpreting the defect-related peaks as a fingerprint of hydrogen incorporation in graphene, and reveals how the method proposed in this work allows one at the same time to achieve an unprecedentedly highly deuterated sample and an unprecedentedly clean and low-defect sample.

## 4. Conclusions

Nanoporous graphene has been efficiently functionalised with deuterium atoms by D${}^{+}$ ion irradiation in controlled conditions, reaching an unprecedented very high uploading (∼50 at.%), with very low defect density. This free-standing D-graphane presents distorted D–C sp${}^{3}$ bonds with respect to the pristine sp${}^{2}$ carbon bonds, as deduced by the lineshape analysis of the C 1s core level spectra. The lattice distortion was confirmed by Raman spectroscopy: the moderate increase of the defect-related peaks suggested very clean D-decoration of graphene. The bond distortion from pristine semimetallic graphene causes the opening of an energy gap, turning D-NPG into a semiconductor, as revealed by the electronic spectral density evolution in the valence band close to the Fermi level. Thus, using CVD growth of NPG and the D${}^{+}$ ion irradiation method is very promising for achieving free standing, low-defect and highly efficient D-graphane in possibly cost-effective and large scale production. The exceptional mechanical properties of graphene accompanied by the obtained semiconducting response of graphane, can pave the way towards pioneering technologies for widespread highly-efficient applications in devices where semiconducting graphane can be combined and engineered with conducting graphene. Furthermore, the achieved D-graphene functionalisation in a safe process opens up new possibilities in view of the use of the heavier tritium isotope as the key element for a futuristic prototype neutrino detector [11].

## Figures and Tables

**Table 1:** Fit values for the C 1s core level of UHV-clean NPG, as-deposited D-NPG and D-NPG after annealing at 150, 250 and 330 ${}^{\circ }$C. Component, binding energy (±0.1 eV, ±0.3 eV for the π-plasmon), asymmetry parameter, full-width at half-maximum (FWHM, ±0.1 eV), relative intensity (peak areas in %, ±5%).

**UHV-clean NPG**				
C 1s component	BE (eV)	Asymm. Param.	FWHM (eV)	Area/Area${}_{tot}$ (%)
sp${}^{2}$	284.6	0.1	1.1	71.3
sp${}^{3}$	285.3	0	1.3	14.8
π-plasmon	291.3	0	4.0	5.3
CO${}_{x}$	286.3	0	1.7	8.6
**as-deposited D-NPG**				
C 1s component	BE (eV)	asymm. param.	FWHM (eV)	Area/Area${}_{tot}$ (%)
sp${}^{2}$	284.6	0.1	1.1	41.3
sp${}^{3}$	285.6	0	1.9	42.8
π-plasmon	291.0	0	4.0	0.3
CO${}_{x}$	287.0	0	2.5	15.6
**D-NPG annealed at 150 ${}^{\circ }$C**				
C 1s component	BE (eV)	asymm. param.	FWHM (eV)	Area/Area${}_{tot}$ (%)
sp${}^{2}$	284.6	0.1	1.1	42.3
sp${}^{3}$	285.6	0	1.7	43.6
π-plasmon	291.1	0	4.0	0.3
CO${}_{x}$	287.0	0	2.2	13.8
**D-NPG annealed at 250 ${}^{\circ }$C**				
C 1s component	BE (eV)	asymm. param.	FWHM (eV)	Area/Area${}_{tot}$ (%)
sp${}^{2}$	284.5	0.1	1.1	43.1
sp${}^{3}$	285.4	0	1.7	43.7
π-plasmon	291.0	0	4.0	0.7
CO${}_{x}$	286.7	0	2.2	12.5
**D-NPG annealed at 330 ${}^{\circ }$C**				
C 1s component	BE (eV)	asymm. param.	FWHM (eV)	Area/Area${}_{tot}$ (%)
sp${}^{2}$	284.5	0.1	1.1	44.3
sp${}^{3}$	285.3	0	1.6	43.6
π-plasmon	291.0	0	4.0	1.9
CO${}_{x}$	286.3	0	2.1	10.2

## Data Availability

Data is contained within the article.

## Abbreviations

The following abbreviations are used in this manuscript:

XPS	X-ray photoelectron spectroscopy
UPS	Ultraviolet photoelectron spectroscopy
NPG	Nanoporous graphene
